# Antero-posterior mandibular position at different vertical levels for mandibular advancing device design

**DOI:** 10.1186/s12903-019-0783-8

**Published:** 2019-05-22

**Authors:** P. Mayoral, M. O. Lagravère, M. Míguez-Contreras, M. Garcia

**Affiliations:** 1 Catholic University of Murcia UCAM, Faculty of Medicine and Dentistry, School of Dentistry and Scientific Committee Orthoapnea, Flauta Mágica 22, 29006 Málaga, Spain; 2 University of Alberta, Faculty of Medicine and Dentistry, School of Dentistry, ECHA 5-524, 11405-87 Avenue, Edmonton, Alberta T6G 1C9 Canada; 3 Catholic University of Murcia UCAM, Faculty of Medicine and Dentistry, School of Dentistry, Madrid, Spain; 4 University of Malaga, Faculty of Engineering and Scientific Committee Orthoapnea, Flauta Mágica 22, 29006 Málaga, Spain

**Keywords:** Obstructive sleep apnea. Mandibular advancement device. Oral appliance design. Mandibular position, Vertical opening. Mandibular protrusion

## Abstract

**Introduction:**

Mandibular Advancement Devices (MAD) have been reported to be an alternative treatment to CPAP in moderate to severe obstructive sleep apnea (OSA) cases. The design of MAD has a major influence on its success rate on the patient, and design features that have an influence on efficacy, tolerance, and compliance. The aim of this study was to determine the range of mandibular protrusion at different vertical points; 2, 5, 8 and 11 mm in a young adult population.

**Methods:**

Fifty two students aged 19 to 23 years (mean 21.3 ± 1.7; 29 females and 23 males), with full permanent dentition participated in the study. The absolute range of maximal mandibular protrusion and retrusion was measured (mm) with the use of the George Gauge. Descriptive statistics, ANOVA and paired t-test using SPSS were used.

**Results:**

Range of mandibular advancement was possible to be determined for the 4 levels of vertical opening with the gauges: 2 mm fork mean mandibular advance 13,10 mm ± 0.604; 5 mm mean 11.98 mm ± 1.075; 8 mm mean 11.20 mm ± 1.369; 11 mm mean 9.87 mm ± 1.886. No significant differences were found between class I, II, and III.

**Conclusions:**

There is an impact of increased inter-incisal distance of effective mandibular protrusion when constructing a MAD. As vertical dimension increases the mandible rotates posteriorly and places itself in a more retrusive location, and the range of mandibular advancement reduces (0.3 mm for every 1 mm of vertical increase).

## Introduction

One of the symptoms of Obstructive sleep apnea (OSA) is the presence of recurrent episodes of partial/complete collapse of the upper airway [1]. This may lead to a fragmentation of the sleep pattern, a decrease in oxygen saturation and a partial pressure rise of CO_2_ in the blood [2]. The arousals and the nocturnal hypoxemia can cause excessive daytime sleepiness, loss of concentration, hypertension and atherosclerosis [3]. In more severe cases, OSA may lead to stroke and heart failure, resulting in an increased prevalence of cardiovascular morbidity and mortality [4].

A type of treatment for OSA consists of the use of mandibular advancement devices (MAD) [1, 5]. These appliances keep the mandible in a forward/protruded position during sleep increasing the width of the airway and reducing its collapsibility [6]. Even though there are conflicting reports on the success rate of these appliances [1] [7], MAD have been reported to be an alternative treatment to Continuous Positive Airway Pressure (CPAP) in moderate to severe OSA cases [6, 8].

It should be noted that the range of success of MAD is limited and can present a high variability between individuals [9]. Clinical evidence over the success rate of MAD are not conclusive thus more research is needed to be able to determine its predictability [10, 11].

The design of the MAD has a major influence on its success rate on the patient [12], and the design features have an influence on efficacy, tolerance, and compliance [13, 14].

There are two important aspects involved in making MAD which are the amount of vertical opening and the degree of mandibular protrusion. In general terms, the more advancement of the mandible, the better treatment results which should be balanced with the potential appearance increase of side effects [1].

Protrusion has been reported to happen by the body translation of the condyles [15]. During protrusion, the condyle translates following the contour of the articular eminence being affected by the steepness of the posterior slope of it [15]. Increasing the vertical dimension causes posterior rotation of the mandible with two adverse effects: possible reduction of maximum protrusive capacity and an increased posterior position of the mandible.

The use of incorrectly designed MAD which give an inappropriate level of protrusion for example short protrusion will not produce an effective reduction of the Apnea Hiponea Index (AHI) [16] and minimal change in total pharyngeal area [17]. If the MAD cause a long protrusion in the patients, it can cause temporomandibular joint issues and negative occlusal changes [18] which limit the effectiveness of the appliance and treatment overall. It is worth noting that an increased vertical mouth opening has an adverse effect on upper airway patency in the majority of OSA patients [19].

The purpose of the study was to determine the impact of increased vertical dimension on the range of protrusion of the mandible. Few authors have studied the range of protrusive movements [20, 21], but none at different vertical levels. Since MAD are constructed at various vertical levels, the aim was to determine the range of mandibular protrusion at different vertical points; 2, 5, 8 and 11 mm in a young adult population. This information will help in determining a guide for construction and application of a MAD appliance.

## Methods

Fifty-two students of Dentistry at the Universidad Alfonso X Madrid, aged 19 to 23 years (mean age 21.3 SD 1.7; 29 females and 23 males) agreed to participate in this study. All subjects were asymptomatic for temporomandibular disorders, according to the Research Diagnostic Criteria/ Temporomandibular Disorders RDC/TMD, RDC /TMD [13]. This study was approved by the ethical review board of Universidad Alfonso X Madrid UAX 2016–021 and written consent was obtained from all participants. All patients had full permanent dentition up to the second molar. None had previous maxillofacial surgery nor TMJ symptomology.

Each subject had a lateral cephalometric radiograph done at the start of the study with profile and frontal extraoral photos. Lateral cephalograms were landmarked using the Kinovea software (Kinovea, France). X and Y coordinates were determined for each landmark. Distances and angles were measured and placed in an excel worksheet. Table 1 lists the landmarks with respective definitions used. Table 2 lists distances and angles measured on each radiograph.

**Table 1:** Cephalometric landmarks used in the study

Landmark	Definition
A-point (A)	Deepest point of the maxillary base between the anterior nasal spine and the alveolar crest
B-Point (B)	Deepest point in the concavity of the anterior border of the symphysis
6 s	Mesiobuccal cusp of the upper first molar
Condyle (Co)	The center point of the condyle
Incisor superior (IS)	The most inferior anterior point of the incisal edge of the maxillary incisor
Incisor inferior (II)	The most superior anterior point of the incisal edge of the mandibular incisor
Nasion (N)	Most anterior superior point at the intersection of the nasal bone and the nasofrontal suture in the midsagittal plane
Sella (S)	Center of sella turcica

**Table 2:** Measurements of the study

Measurement	Definition
Angles	
SNA	Angle formed by the planes S-N and N-A
SNB	Angle formed by the planes S-N and N-B
ANB	Angle formed by the planes N-A and N-B
Especial Measurements	
galgarx2	Distance from tip of upper incisor (landmark IS), parallel to the occlusal plane at 2 mm, to arch with its center in the condyle (landmark Co) and having the curve pass through the inferior incisor (landmark II)
galgarx5	Same as before at 5 mm
galgarx8	Same as before at 8 mm
galgarx11	Same as before at 11 mm

**Fig. 1:**
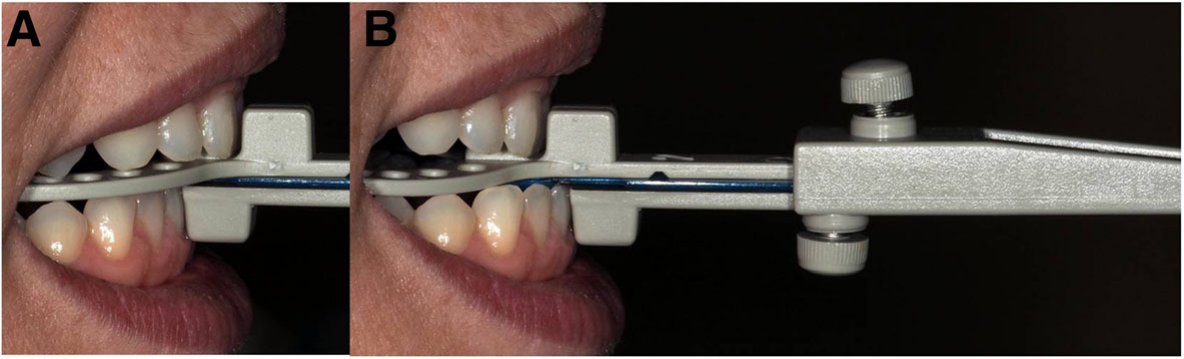
George gauge maximum retrusion and maximum protrusion. Shows how the absolute range of maximal mandibular retrusion (**a**) and protrusion (**b**) was measured (in mm) with the use of the George Gauge.

**Fig. 2:**
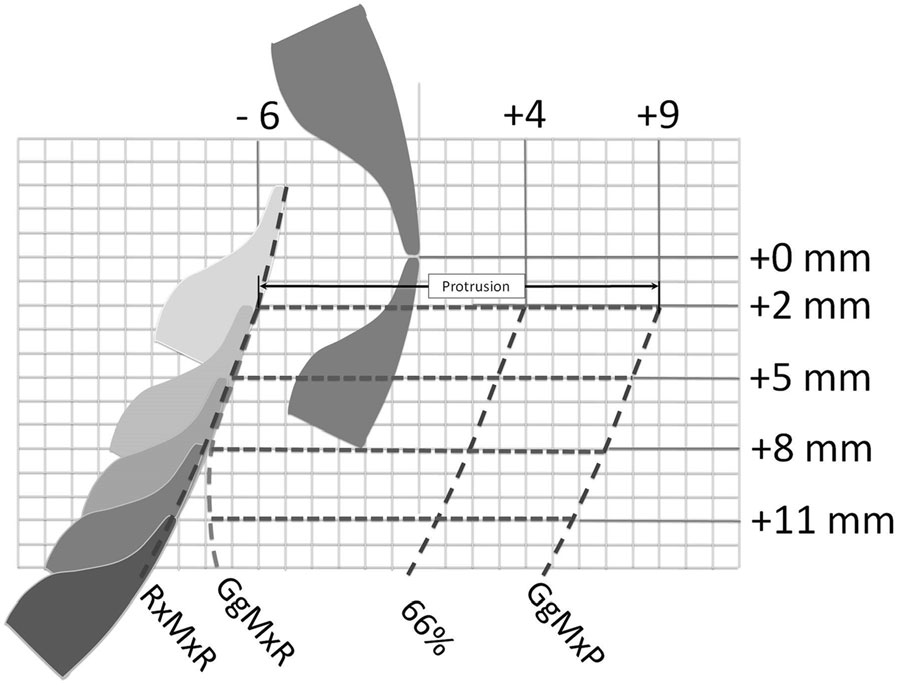
Position of lower incisor in relation to upper incisor. RxMxR shows the maximum retrusion calculated with the radiograph. GgMxR shows the maximum retrusion obtained with the George Gauge. 66% shows the position of the lower incisor with 66% of maximum protrusion. GgMxP shows the maximum protrusion obtained with the George Gauge. Values obtained with the George Gauge with 2 mm of vertical opening are shown on the figure; −6 mm for maximum retrusion; + 9 mm for maximum protrusion, + 4 for 66% of total advance.

When taking measurements after doing the different vertical openings, patients were asked to sit straight in the dental chair. The absolute range of maximal mandibular protrusion and retrusion was measured (in mm) with the use of the George Gauge (Great Lakes Orthodontics, Ltd., New York, USA) [22] (Fig. 1). Then, the principal investigator took measurements of the maximum protrusion range with 4 different George Gauge bite forks (2 mm, 5 mm, 8 mm, and 11 mm of interincisal vertical opening) (Table 3). By definition, maximum retrusion was considered when the lower incisors were located behind the upper incisors (negative value) and maximum protrusion was considered when the lower incisors were located in front of the upper incisors (positive value) (Fig. 2).

**Table 3:** Total mandibular advance in milimeters

Landmark	Definition
A-point (A)	Deepest point of the maxillary base between the anterior nasal spine and the alveolar crest
B-Point (B)	Deepest point in the concavity of the anterior border of the symphysis
6 s	Mesiobuccal cusp of the upper first molar
Condyle (Co)	The center point of the condyle
Incisor superior (IS)	The most inferior anterior point of the incisal edge of the maxillary incisor
Incisor inferior (II)	The most superior anterior point of the incisal edge of the mandibular incisor
Nasion (N)	Most anterior superior point at the intersection of the nasal bone and the nasofrontal suture in the midsagittal plane
Sella (S)	Center of sella turcica

*N* Number of patients

*SD* Standard Deviation

5 patients had overbite of less than 2 mm which didn’t allow to use the fork of 2 mm

**Fig. 3:**
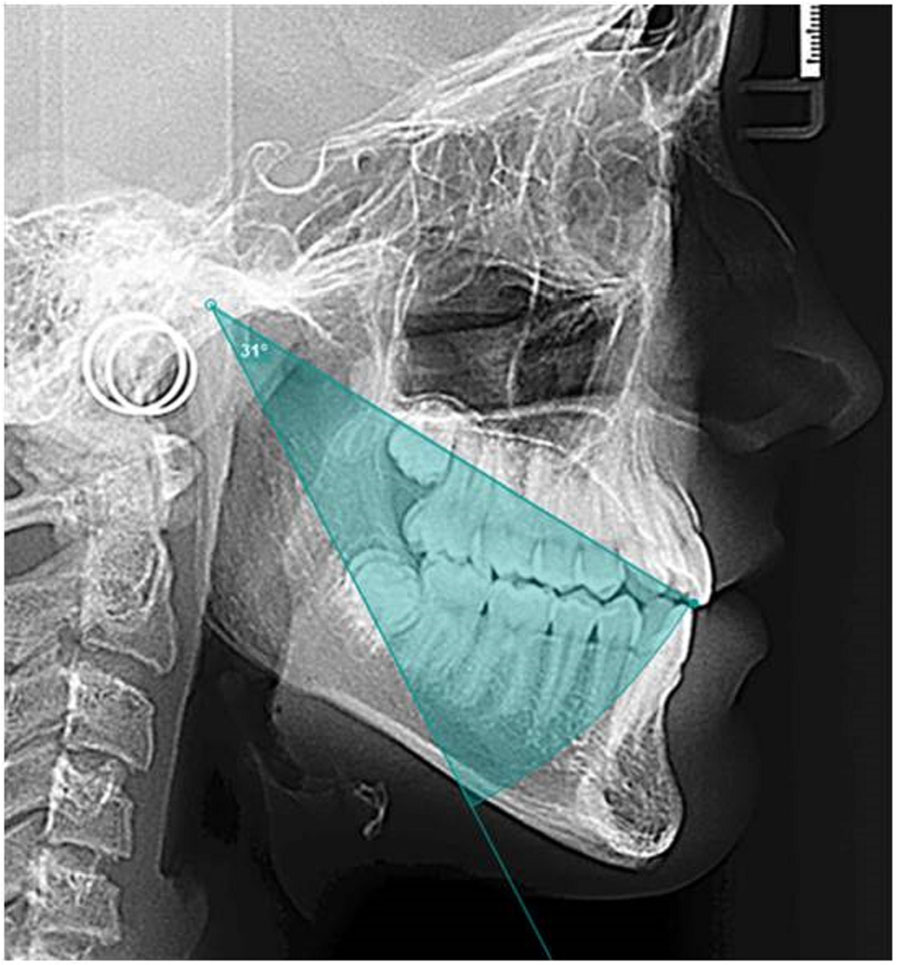
Rotational movement of the mandible. Shows how the rotational movement of the mandible was calculated in each patient with the cephalometric radiography. The rotational movement is an arch with its center in the condyle (landmark Co) and having the curve pass through the inferior incisor through the line connecting the condyle and inferior incisor (Co-II).

Maximun retrussion was also calculated using the cephalometric radiograph. As Posselt mentions [23] the posterior curve represents the motion limit of the mandible in its most posterior location. This occurs as a rotational movement of the mandible in its hinge in the center of the condyles [24]. This movement is represented as an arch with its center in the condyle (landmark Co) and having the curve pass through the inferior incisor through the line connecting the condyle and inferior incisor (Co-II) (Fig. 3).

**Fig. 4:**
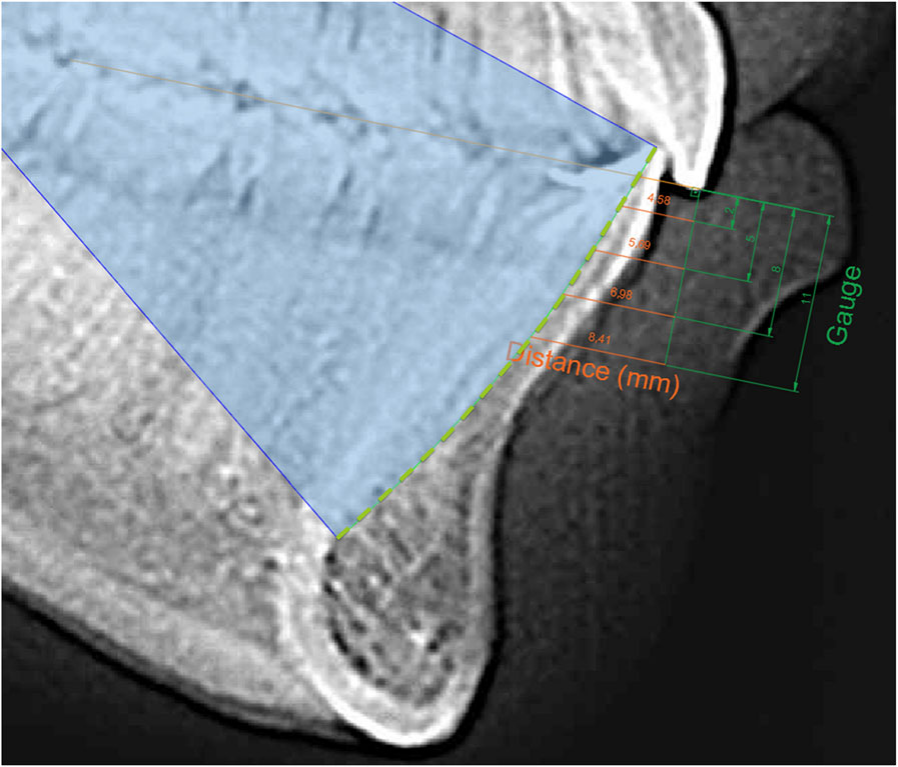
Planes at different vertical opening levels. Shows the curve of the rotational movement, discontinuous curve, the maxillary occlusal plane used with the Gauge, and the parallel lines to the maxillary occlusal plane representing Gauges 2,5,8 and 11 mm of opening.

Once the posterior curve was determined, discontinuous curve in Fig. 4, the maxillary occlusal plane was defined as the movement plane of the mandible using the Gauge. This was to compare the measurements obtained from the Gauge to the ones from the radiograph. Lastly, parallel lines to the maxillary occlusal plane were drawn representing Gauges 2,5,8 and 11 of opening. Then the distance was measured between the incisor position and the theoretic posterior curve as seen in Fig. 4.

Distances were calculated using the coordinates obtained from each landmark using the Euclidean distance. Samples were also categorized into skeletal relationship Class I, II or III based on their ANB measurement obtained from the cephalometric radiographs. (ANB: Class III <1degree, Class I > 1 and < 5, Class II > 5. Table 3 shows the distribution of the sample with respect to Class, age and gender.

Ten radiographs were randomly selected and landmarked three times leaving one week in between trials to calculate reliability of the measurements. Interclass Correlation Coefficient was used to calculate the reliability as well as the measurement error for each landmark in each coordinate. Descriptive statistics were used as well as ANOVA and paired t-test using SPSS (version 24, IBM, New York, USA).

## Results

All landmarks presented excellent reliability values with the lowest landmark being 0.97 (CI 95% 0.94;0.99) in the A point y-axis. The majority of landmarks had an average of > 1 mm error in all coordinates.

**Fig. 5:**
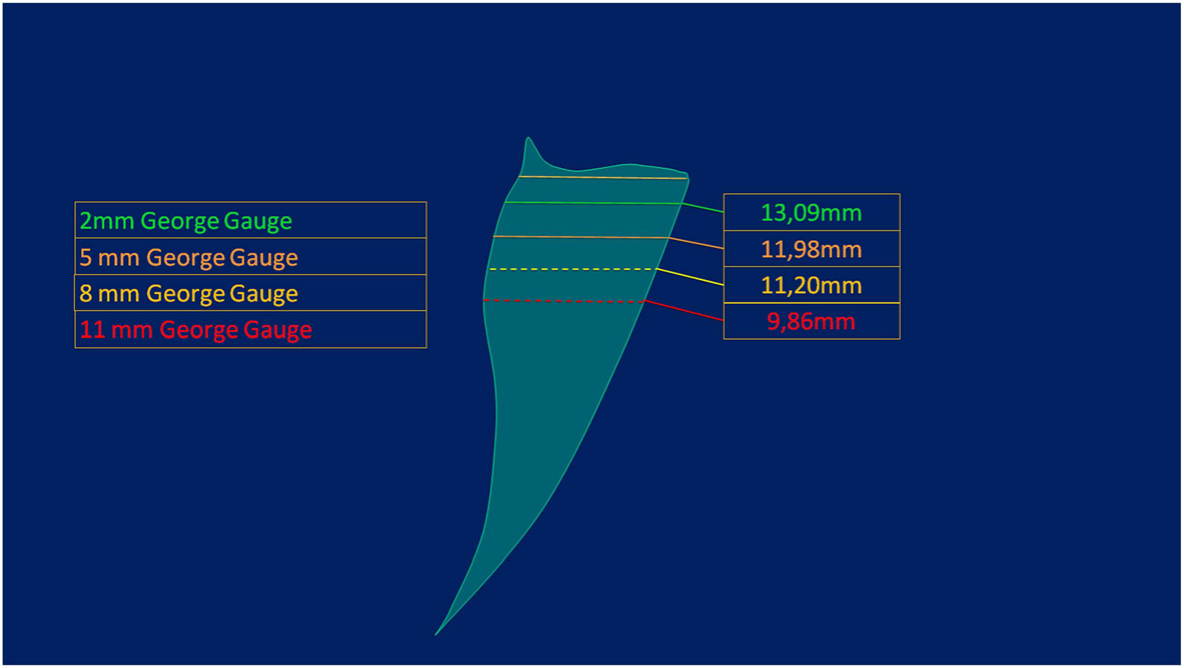
Normal range of maximum protrusion at different vertical opening levels. Shows the range of maximum protrusion obtained in our study for the different vertical opening levels and with it, constructed the Posselt diagram of border movements of the mandible.

Once reliability was determined, all cephalograms were landmarked and measured. Range of mandibular advancement was determined for the 4 levels of vertical opening with the gauges (Fig. 5). It was found that as the vertical opening increased, the range of anteroposterior movement of the mandible decreased. (Table 3) Patient were diagnosed using skeletal relationships obtained from the radiographs to group them into Class I, II or III. No significant differences were found between class I, II, and III, although patients classified as Class III had a larger range of motion compared to Class II patients.

Figure 2 illustrates that as the vertical dimensions increased to 8 mm, the mandibular position became more retrusive, but when it is increased to 11 mm, the retrusion diminished.

When viewing the values obtained by the Gauge from the Gauge of George and the values from the radiographs, it was found that both presented a positive correlation. The higher the gauge, the greater the value obtained as seen in Table 4.

**Table 4:** Maximum retrusion on Radiograph vs George Gauge

	Vertical opening	N	Min	Max	Mean	SD
Radiograph	2 mm	52	−9,28	−1,54	−4.87	1.557
	5 mm	52	−10,63	−2,96	−6.07	1.570
	8 mm	52	−12,09	−4,23	−7.36	1.610
	11 mm	52	−13,66	−5,64	−8.76	1.667
George Gauge	2 mm	47	−8,50	−3,00	−5.48	1.339
	5 mm	52	−9,00	−3,00	−5.45	1.311
	8 mm	52	−9,00	− 3,00	−6.27	1.293
	11 mm	52	−10,00	−3,00	−6.10	1.704
		Paired Samples Correlations		Paired Differences		
		Correlation	Sig.	Mean	SD	Sig. (2-tailed)
Radiograph & George Gauge	2 mm	0.603	0.000	1.26465	0.18447	0.026
	5 mm	0.565	0.000	1.35309	0.18586	0.003
	8 mm	0.490	0.000	1.51619	0.21231	0.000
	11 mm	0.349	0.011	1.99586	0.27678	0.000

*N* Number of patients

*SD* Standard Deviation

5 patients had overbite of less than 2 mm which didn’t allow to use the fork of 2 m

## Discussion

To our knowledge, this is the first study in which the maximum protrusive capacity of an adult population was measured with George Gauge with four levels of vertical increase. Two factors that can affect the mandibular advancement and the efficiency of the MAD have been studied. With an increase in the vertical dimension, the range of mandibular advancement reduces (0.3 mm for every 1 mm of vertical increase up to 8 mm of interincisal distance). Also, with an increase in the vertical dimension, the mandible rotates posteriorly and places itself in a more retrusive location. To compensate for this vertical increase, it is necessary to increase the amount of mandibular advancement when constructing the MAD (Fig. 2). The rationale for the present study was to determine the range of advancement at four different vertical levels in a young adult population. As a result from this, it was found that the range of advancement also produces posterorotation.

It is important to identify the maximum protrusion in a young adult since this serves as a reference for the maximum protrusion that can be accomplished using MAD. The MAD are made so that mandibular advancement can occur that will open and keep the airway open when the patient is sleeping. Knowing the values of normal range will make it possible to design an appliance that will be effective, tolerated by the patient and will not produce secondary effects.

Few studies have registered maximum protrusions of the mandible and no study was found that correlated it with an increase in the vertical dimension. Children present a protrusion from 5 to 6 mm [25]. Bonjardim et al. [25] analyzed this in children 3–5 years of age and found that the maximum protrusion was 5.67 mm ± 1.76 mm in Class I groups and 6.12 mm ± 1.92 mm in Class II groups. Adolescents have a protrusion of 10.6 mm ± 3.2 [26]. These values are smaller than the ones found in the present study although the present study analyzed older patients.

Adults present a mandibular protrusion on average of 9–11 mm [27–31] with a sample range of variation (5–16 mm) between individuals [28] [30] which are smaller values compared to the present study. Ingervall’s study [28] found in an adult female sample (21 years old) that the maximum distance was 9 mm (range 5 to14mm). Posselt [27] found values of 11 mm (10 to 14 mm) in both females and males. Agerberg [29] also found values of 10 mm for both genders between 18 and 25 years of age.

When designing a MAD, different degrees of mandibular protrusion and vertical opening should be considered but the decision of what is optimal is still questionable. [8, 32, 33].

Protrusion positions with 25, 33, 50, 67, 75, and 100% of the maximum mandibular protrusion have been examined in previous studies [34–46]. The degree of advancement is usually expressed as a % of maximum protrusive capacity or/and in millimeters (mm). Percentage of maximum protrusive capacity is used in reference to potential side effects and percentage or millimeters to effectiveness in opening the upper airway.

Mandibular protrussion effects has been researched more than the effect of vertical openings [8, 17, 32, 33]. With that being considered, the more appropriate vertical opening in terms of treatment is still debatable [8, 17, 33]. Some degree of vertical opening is unavoidable because of device design features and an appropriate degree of vertical opening is desirable for optimizing the forward displacement of the mandible [47]. The amount of vertical opening used in different devices is given in millimeters (range 1–14 mm) [8, 34, 38–40, 42–44]. Although studies have shown that appliances with both an increased [38, 48, 49] or minimal [49, 50] vertical dimension are effective, physiologic evidence suggest that the vertical dimension should be kept to a minimum to optimize treatment outcome [24]. Our results help to understand the findings of Piskin where the most increase happened in the pharynx with a splint having the least vertical occlusion with the highest degree of protrussion [17]. Furthermore, an increased vertical mouth opening has an adverse effect on upper airway patency in the majority of OSA patients [19]. For this reason, the amount of bite opening should be minimized which improves patient tolerance and positive effect on upper airway [1].

In the present study, similar to the Piskin study, the inclusion of full permanent dentition patients with unaffected vertical occlusion was to confirm that the vertical opening was functional and not because of loss of teeth [17].

No significant differences were found between patients skeletal class I, II, and III for the range of anteroposterior movement of the mandible (Table 3) for 2 mm interincisal vertical opening. Although patients classified as Class III had a greater range of motion compared to Class II patients for 5, 8 and 11 mm. Patients with skeletal Class II have shorter or more retrusive mandibles than Class III, and this makes Class II subjects to have a more retrusive path of aperture which could explain the smaller range of anteroposterior movement.

When viewing the values of maximum retrusion obtained from the Gauge of George and the values from the radiograph, it was found that both presented a positive correlation for 2, 5 and 8 mm but not for 11 mm (Table 4). When analyzing the data, it was found that as the vertical dimensions increased from 2 to 8 mm, the mandibular position becomes more retrusive. However, when it increased to 11 mm, the retrusion diminishes as observed in Fig. 2. This suggests that the point where pure rotation ends and rotation plus translation starts is about 8 mm of interincisal distance and this is consistent with previous studies [24] that stated that the translation and rotation occurred simultaneously from early opening.

Mandibular movement presents a difficult pattern to describe [51], however, the results of our study can partially be explained by the concept of the functionality of mandibular movement and therefore necessary to consider when making a MAD.

The four main areas of variability among MAD are freedom of mandibular movement, amount and rigidity of dental coverage, amount of mandibular advancement and amount of bite opening. Before selecting the parameters of the construction bite, the dentist must carefully weigh the advantages and disadvantages of each millimeter of mandibular forward translation and each degree of downward rotation [12].

One limitation found in the present study is that control for ethnicity was not achievable since the multiethnic backgrounds of the patients made it hard to focus on one specific group. For future studies, comparisons between ethnic groups would be interesting since the overall size of the mandible may play an important role.

Following the results from this study, it is suggested to limit the aperture since each mm of opening creates a posterior rotation limiting the protrussion.

## Conclusion

There is an impact of increased inter-incisal distance of effective mandibular protrusion when constructing a MAD. As vertical dimension increases the mandible rotates posteriorly and places itself in a more retrusive location. With an increase in the vertical dimension, the range of mandibular advancement is reduced (0.3 mm for every 1 mm of vertical increase up to 8 mm of interincisal distance). It is recommended to limit the amount of opening or increase in the vertical dimension in patients that would use MAD.

Before selecting the parameters of the construction bite, the dentist must carefully weigh the advantages and disadvantages of each millimeter of mandibular forward translation and each degree of downward rotation.

## Abbreviations

AHI: Apnea Hipopnea Index
CPAP: Continuous Positive Airway Pressure
MAD: Mandibular Advancement Device
OSA: Obstructive Sleep Apnea
RDC: Research Diagnostic Criteria
TMD: Temporomandibular Disorders
